# Performance evaluation of tuberculosis control in Brazilian municipalities

**DOI:** 10.11606/s1518-8787.2022056004020

**Published:** 2022-06-07

**Authors:** Priscila Fernanda Porto Scaff Pinto, Beatriz Pinheiro Schindler dos Santos, Camila Silveira Silva Teixeira, Joilda Silva Nery, Leila Denise Alves Ferreira Amorim, Mauro Niskier Sanchez, Mauricio Lima Barreto, Julia Moreira Pescarini

**Affiliations:** I Fundação Oswaldo Cruz Centro de Integração de Dados e Conhecimentos para a Saúde Salvador BA Brasil Fundação Oswaldo Cruz. Centro de Integração de Dados e Conhecimentos para a Saúde. Salvador, BA, Brasil; II Universidade Federal da Bahia Instituto de Saúde Coletiva Departamento de Saúde Coletiva I Salvador BA Brasil Universidade Federal da Bahia. Instituto de Saúde Coletiva. Departamento de Saúde Coletiva I. Salvador, BA, Brasil; III Universidade Federal da Bahia Instituto de Matemática Departamento de Estatística Salvador BA Brasil Universidade Federal da Bahia. Instituto de Matemática. Departamento de Estatística. Salvador, BA, Brasil; IV Universidade de Brasília Faculdade de Ciências da Saúde Departamento de Saúde Coletiva Brasília DF Brasil Universidade de Brasília. Faculdade de Ciências da Saúde. Departamento de Saúde Coletiva. Brasília, DF, Brasil; V London School of Hygiene & Tropical Medicine Faculty of Epidemiology and Population Health London UK London School of Hygiene & Tropical Medicine. Faculty of Epidemiology and Population Health. London, UK

**Keywords:** Tuberculosis, prevention & control, Outcome and Process Assessment, Health Care, Program Evaluation, Ecological Studies

## Abstract

**OBJECTIVE:**

To evaluate the performance of tuberculosis control in Brazilian municipalities.

**METHODS:**

This is an ecological study on Brazilian municipalities that notified at least four new cases of tuberculosis, with a minimum of one new case of pulmonary tuberculosis between 2015 and 2018. The municipalities were stratified according to the population in < 50 thousand, 50–100 thousand, 100–300 thousand, and > 300 thousand inhabitants, and the k-means method was used to group them within each population range according to the performance of six indicators of the disease.

**RESULTS:**

A total of 2,845 Brazilian municipalities were included, comprising 98.5% (208,007/211,174) of new tuberculosis cases in the period. For each population range, three groups (A, B, and C) of municipalities were identified according to the performance of the indicators: A, the most satisfactory; B, the intermediates; and C, the least satisfactory. Municipalities in group A with < 100 thousand inhabitants presented results above the targets for laboratory confirmation (≥ 72%), abandonment (≤ 5%), and cure (≥ 90%), and comprised 2% of new cases of the disease. Conversely, municipalities of groups B and C presented at least five indicators with results below the targets – HIV testing (< 100%), contact investigation (< 90%), directly observed therapy (< 90%), abandonment (> 5%), and cure (< 90%) –, and corresponded to 66.7% of new cases of tuberculosis. In group C of municipalities with > 300 thousand inhabitants, which included 19 of the 27 capitals and 43.1% of new cases of tuberculosis, the lowest percentages of contact investigation (mean = 56.4%) and directly observed therapy (mean = 15.4%) were verified, in addition to high abandonment (mean = 13.9%) and low coverage of primary health care (mean = 66.0%).

**CONCLUSIONS:**

Most new cases of tuberculosis occurred in municipalities with unsatisfactory performance for disease control. Expanding the coverage of primary health care in these places can reduce abandonment and increase the contact investigation and directly observed therapy.

## INTRODUCTION

Brazil is among the thirty countries with the highest number of tuberculosis cases^1^. In 2020, 66,800 new cases of the disease were notified and the incidence was 31.6 per 100 thousand inhabitants, values lower than expected due to the covid-19 pandemic, considering that in 2019 the incidence was 37.4 per 100 thousand inhabitants and mortality was 2.2 per 100 thousand inhabitants^2^. The *Plano Nacional pelo Fim da Tuberculose como Problema de Saúde Pública* (National Plan for Ending Tuberculosis as a Public Health Issue) was launched in 2017 with targets to reduce the incidence to less than 10/100 thousand inhabitants and mortality to less than 1/100 thousand inhabitants by 2035^3^.

The evaluation and monitoring of health indicators have emphasized the importance of the Brazilian Unified Health System (SUS) at all levels of health care^4^. The evaluation of operational indicators of tuberculosis guides decision-making and directs health actions and policies to tackle this health issue^2^. In 2019, the indicators of cure (70.1%) and abandonment (12%)^2^ were below the expected for disease control^5^, considering that the World Health Organization recommends that the cure rate be at least 90% and up to 5% as for abandonment^6^. These treatment outcomes may be influenced by the quality of tuberculosis control programs^7^.

A previous evaluation of the performance of tuberculosis control initiatives performed between 2001 and 2003 identified that Brazilian municipalities with good performance were smaller in population size, and may or may not present a high performance of directly observed therapy (DOT). Conversely, municipalities with regular and moderate performance are medium- or large-sized, have high abandonment and higher incidences for tuberculosis and/or Aids^8^. A recent evaluation of tuberculosis control programs incorporated socioeconomic, epidemiological, and operational indicators to the classification of municipalities by priority of programmatic action of the Brazilian Ministry of Health, suggesting the adoption of intersectoral actions according to the incidence of the disease as per the specificity of each local reality^9^.

However, the classifications solely carried out by operational indicators of the disease allow evaluating tuberculosis care regardless of the incidence, which is useful, because it allows prioritizing programmatic actions on a local scale, aiming at a more effective control of the disease. Thus, the objective of this study was to evaluate the performance of Brazilian municipalities according to the operational indicators of tuberculosis and to describe/investigate its relationship with epidemiological and contextual indicators between 2015 and 2018.

## METHODS

### Study Design and Sources of Information

Ecological study whose units of analysis were the Brazilian municipalities classified based on operational indicators of tuberculosis between 2015 and 2018. A period of four years was chosen, aiming to mitigate the variations of indicators that could occur if only one year was chosen. The choice was also made considering that 2018 was the last year in which the treatment outcomes were still available in the data. The records of tuberculosis and Aids were extracted from the *Sistema de Informação de Agravos de Notificação* (Sinan – Notifiable Diseases Information System) (2015–2018) on October 17, 2020. The annual population estimates (2015–2018) and the unemployment rate per municipality among people ≥ 16 years old for 2010 were obtained from the *Instituto Brasileiro de Geografia e Estatística* (IBGE - Brazilian Institute of Geography and Statistics). The percentage of primary health care (PHC) coverage derives from the database *Banco e-Gestor AB* (e-Manager Database PHC) (December 2018).

### Study Population

Brazilian municipalities that notified Sinan of at least four new cases of tuberculosis in the period (2015–2018) were included in the study, being at least one new case of pulmonary tuberculosis, a criterion used to include those that, on average, have reported a new case of tuberculosis per year and to avoid zero-inflated data and estimation of indicators with high variation due to a few cases. The new case of tuberculosis was defined as the person notified to Sinan with the active disease, regardless of the clinical classification, who has never undergone anti-tuberculosis treatment or did so for less than 30 days; and, as a new case of pulmonary tuberculosis, the person notified to Sinan with the active disease, whose clinical classification was pulmonary, that is, when the bacillus affects the lungs^10^.

### Variables

Altogether, in the period from 2015 to 2018, the eleven operational indicators of tuberculosis of the *Plano Nacional pelo Fim da Tuberculose como Problema de Saúde Pública* (National Plan for Ending Tuberculosis as a Public Health Issue) were estimated. For this study, five indicators were excluded because they presented low coverage, that is, impossibility of estimation for at least 52.8% of the municipalities: number of tuberculosis cases notified as postmortem; sputum culture performance among cases of pulmonary tuberculosis retreatment; conducting sensitivity test among cases of pulmonary tuberculosis retreatment with positive culture; antiretroviral therapy among new cases of tuberculosis-HIV coinfection; and cure of new cases of multidrug-resistant tuberculosis. Thus, the six indicators evaluated were: laboratory confirmation among new cases of pulmonary tuberculosis; contact investigation of new pulmonary tuberculosis cases with laboratory confirmation; testing for the human immunodeficiency virus (HIV) among new cases of tuberculosis; DOT for new cases of pulmonary tuberculosis; treatment abandonment among new cases of pulmonary tuberculosis with laboratory confirmation; and cure of new cases of pulmonary tuberculosis with laboratory confirmation^5^.

Among the epidemiological indicators, the annual means of incidence and mortality for all types of tuberculosis and the Aids detection rate were estimated. Among the contextual indicators, that is, those that can influence the occurrence of the disease and the performance of programmatic actions^11^, the unemployment rate and coverage of primary health care were included for being associated with the incidence of tuberculosis in previous evaluation studies^9,12^.

### Analysis

A descriptive analysis of the operational indicators of tuberculosis was performed in each municipality using the estimation of means and respective standard deviations. The means were chosen because they are the most common measure of central tendency for tuberculosis indicators; they are close to the medians; they consider all values of the data set; and they are comparable with the k-means method and other evaluations such as the present study. The municipalities were stratified in the following population ranges: ≤ 50 thousand inhabitants; 50 to 100 thousand inhabitants; 100 to 300 thousand inhabitants; and > 300 thousand inhabitants. For each population range, the municipalities were grouped according to the performance of the operational indicators of tuberculosis by the non-hierarchical k-means method, which defines the total intragroup variation as the sum of squares of the Euclidean distances between the points and their respective centroids (within sum of squares – WSS). The k-means method identifies a k number of groups with similar characteristics, that is, whose distances between the means of a variable are minimum for the members of the same group and are maximum in relation to the other groups^13^. A total of 200 initial seeds were used to ensure the stability of the results. The value of k was defined by the elbow method, which draws a WSS chart according to the number of groups, allowing to identify an appropriate value, that is, the number of groups in which the data can be divided so that the groups are sufficiently distinct from each other^14^.

The operational indicators of tuberculosis were interpreted according to compliance or not with the targets and/or recommendations, namely: ≥ 72% for laboratory confirmation^10^, ≥ 90% for contact investigation^15^, 100% for HIV testing^10^, ≥ 90% for performing DOT^16^, ≤ 5% for abandonment^6^, and ≥ 90% for cure^15^.

After grouping the municipalities according to the performance of the operational indicators of tuberculosis, a descriptive analysis of the epidemiological indicators of tuberculosis and Aids was performed, in addition to analyzing the contextual indicators for each verified group based on the means and respective standard deviations, medians and respective interquartile ranges.

To test whether the groups generated by the k-means method were sensitive to the classification method, the groups were re-estimated and described by latent profile analysis (LPA). A diagonal covariance matrix was adopted, with different variances per profiles, considering between two and eight latent profiles. The Akaike Information Criterion (AIC), Bayesian Information Criterion (BIC), and entropy measures were evaluated. The latent profiles for each population range were characterized by means, standard deviations, and correlation matrix^17^.

The analyses were performed in the R Studio program linked to R, version 4.0.3, with the aid of tidyverse, factoextra, NbClust, and tidyLPA libraries.

### Ethical Considerations

The study used administrative data of open access and aggregated by municipalities, thus exempt from variables that enable identifying people with tuberculosis and, therefore, no ethical approval was required.

## RESULTS

Between 2015 and 2018, 4,643 of the 5,570 Brazilian municipalities (83.3%) notified new cases of tuberculosis. We included 2,845 (61.3%) municipalities with at least four new cases of tuberculosis and one case of pulmonary tuberculosis in the study. Thus, the study comprised 98.5% (208,007 out of 211,174) of new cases of the disease in this period, of which 46.6% occurred in the Southeast, 25.6% in the Northeast, 11.6% in the South, 11.5% in the North, and 4.6% in the Midwest.

Small-sized municipalities (< 50 thousand inhabitants) represented 19% of the total and 2.1% of new cases of tuberculosis. Small-medium-sized municipalities (50 to 100 thousand inhabitants) (32.9%) accounted for 5.4% of new cases; medium-large-sized municipalities (100 to 300 thousand inhabitants) (33%) accounted for 12.9% of new cases; and large-sized municipalities (> 300 thousand inhabitants) (15.1%) accounted for 79.6% of new cases of tuberculosis (Table 1).

**Table 1:** Distribution of municipalities and new cases of tuberculosis, according to population range and groups of performance of operational indicators of the disease, generated by cluster analysis, Brazil, 2015 to 2018.

Population range	Groups of performance						Total	
	A		B		C			
	Municipalities	New cases of tuberculosis	Municipalities	New cases of tuberculosis	Municipalities	New cases of tuberculosis	Municipalities	New cases of tuberculosis
	n = 1,351	n = 69,236	n = 1,071	n = 43,488	n = 423	n = 95,283	n = 2,845	n = 208,007
	n (%)	n (%)	n (%)	n (%)	n (%)	n (%)	n (%)	n (%)
< 50 thousand inhab.	292 (21.6)	2.667 (3.9)	218 (20.4)	1.570 (3.6)	30 (7.1)	138 (0.14)	540 (19.0)	4,375 (2.1)
50–100 thousand inhab.	460 (34.0)	5.888 (8.5)	375 (35.0)	4,459 (10.3)	100 (23.6)	927 (1.0)	935 (32.9)	11,274 (5.4)
100–300 thousand inhab.	433 (32.1)	11,819 (17.1)	333 (31.1)	10,417 (24.0)	174 (41.1)	4.616 (4.8)	940 (33.0)	26,852 (12.9)
> 300 thousand inhab.	166 (12.3)	48,862 (70.6)	145 (13.5)	27,042 (62.2)	119 (28.1)	89,602 (94.0)	430 (15.1)	165,506 (79.6)

**Figure 1:**
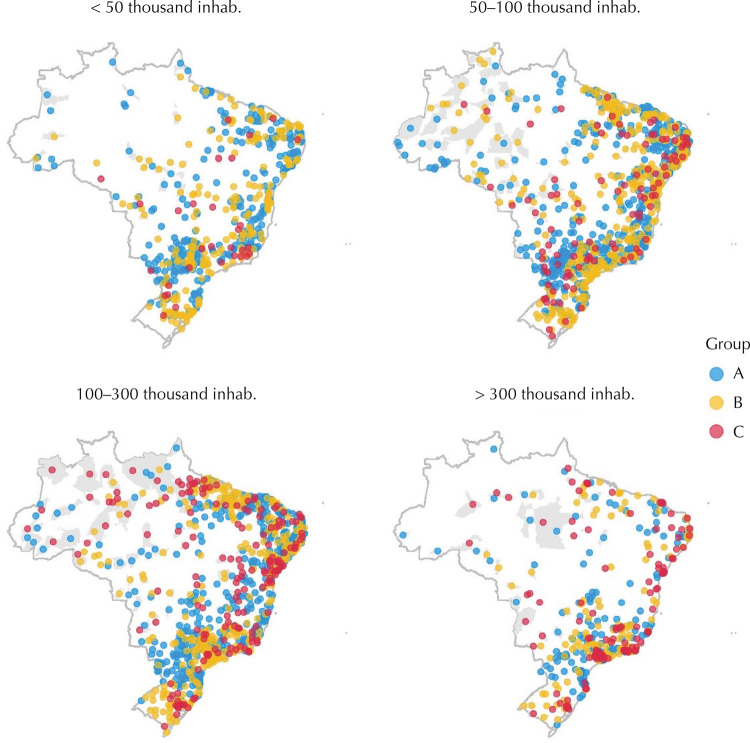
Distribution of Brazilian municipalities included in the study, according to population range and groups of performance of operational indicators of tuberculosis, generated by cluster analysis, Brazil, 2015 to 2018.

Note: The gray area corresponds to the municipalities included in the study.

**Figure 2:**
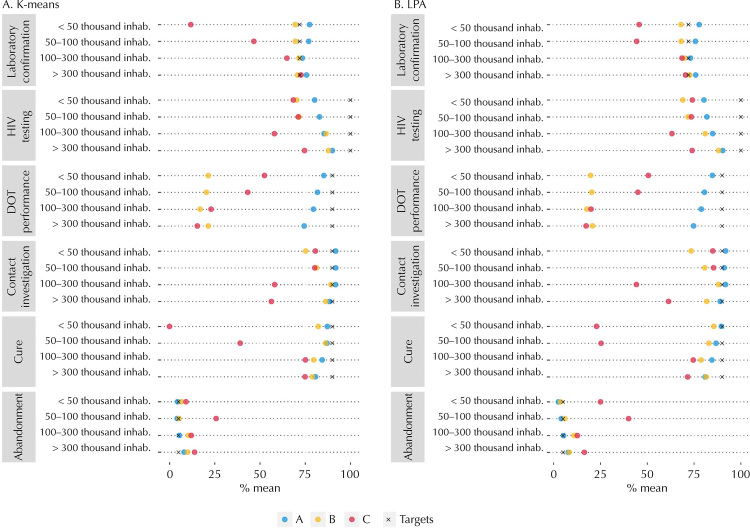
Description of the operational indicators of tuberculosis, according to population range and groups of performance of operational indicators of the disease, generated by: a) cluster analysis (k-means); and b) latent profile analysis (LPA) method, Brazil, 2015–2018.

For each population range, the elbow method indicated a classification with a number of groups ranging from two to four. Thus, we opted for three groups that were named A, B, and C (Figure 1). Overall, the municipalities of group A obtained more satisfactory indicators; those of group B, intermediate indicators; and those of group C, less satisfactory indicators, thus establishing the size of the municipality. Laboratory confirmation was above the target (≥ 72%) in all municipalities of group A and in municipalities with > 300 thousand inhabitants of group C (45.2% of new cases). The contact investigation was above the target (≥ 90%) in municipalities with up to 300 thousand inhabitants (33.3% of new cases) of group A. HIV testing, DOT, and cure did not meet the target (< 100%, < 90%, and < 90%, respectively) in any group. Abandonment was above the target (≤ 5%) only in group A of municipalities with < 100 thousand inhabitants (4.1% of new cases) (Figure 2 and Table 2).

Inhab.: inhabitants; HIV: human immunodeficiency virus; DOT: directly observed treatment.

**Table 2:** Description of the operational indicators of tuberculosis, according to population range and groups of performance of operational indicators, generated by cluster analysis, Brazil, 2015 to 2018.

Operational indicators of tuberculosis according to population range	Groups of performance			Total n = 540
	A	B	C	
< 50 thousand inhab.	n = 292	n = 218	n = 30	
	Mean (SD)	Mean (SD)	Mean (SD)	Mean (SD)
LC among new cases of pulmonary tuberculosis (%)	77.4 (22.9)	69.5 (25.1)	11.8 (21.9)	70.6 (28.0)
HIV test in new cases of tuberculosis (%)	80.3 (23.1)	70.3 (27.3)	68.5 (33.5)	75.6 (26.0)
DOT among new cases of pulmonary tuberculosis (%)	85.3 (16.3)	21.4 (18.3)	52.5 (38.3)	57.7 (36.1)
Examination of contacts in new cases of tuberculosis (%)	91.9 (13.6)	75.3 (26.7)	80.7 (30.4)	84.6 (22.4)
Cure among new cases of pulmonary tuberculosis with LC (%)	87.3 (18.0)	82.2 (21.5)	0.0 (0.0)	80.4 (27.4)
Abandonment among new cases of pulmonary tuberculosis with LC (%)	4.5 (11.3)	6.5 (13.4)	9.2 (26.7)	5.6 (13.5)
**50 a 100 thousand inhab.**	**n = 460**	**n = 375**	**n = 100**	**n = 935**
	**Mean (SD)**	**Mean (SD)**	**Mean (SD)**	**Mean (SD)**
LC among new cases of pulmonary tuberculosis (%)	77.0 (19.1)	69.6 (21.0)	46.7 (24.9)	70.8 (22.5)
HIV test in new cases of tuberculosis (%)	83.0 (19.0)	71.6 (24.9)	71.2 (25.6)	77.2 (23.0)
DOT among new cases of pulmonary tuberculosis (%)	81.8 (16.3)	20.4 (17.6)	43.3 (28.8)	53.1 (34.5)
Examination of contacts in new cases of tuberculosis (%)	92.0 (13.8)	81.3 (20.6)	80.3 (22.2)	86.4 (18.6)
Cure among new cases of pulmonary tuberculosis with LC (%)	87.1 (14.6)	86.4 (14.5)	39.1 (24.6)	81.7 (21.7)
Abandonment among new cases of pulmonary tuberculosis with LC (%)	4.2 (8.3)	5.3 (9.2)	25.8 (28.8)	7.0 (14.1)
**100 a 300 thousand inhab.**	**n = 433**	**n = 333**	**n = 174**	**n = 940**
	**Mean (SD)**	**Mean (SD)**	**Mean (SD)**	**Mean (SD)**
LC among new cases of pulmonary tuberculosis (%)	73.5 (17.5)	71.6 (17.8)	64.9 (19.8)	71.2 (18.3)
HIV test in new cases of tuberculosis (%)	85.5 (17.1)	86.6 (13.1)	58.0 (20.2)	80.8 (19.7)
DOT among new cases of pulmonary tuberculosis (%)	79.6 (16.2)	16.9 (15.6)	23.0 (19.6)	46.9 (34.6)
Examination of contacts in new cases of tuberculosis (%)	91.9 (11.3)	89.3 (10.9)	58.2 (22.1)	84.7 (18.8)
Cure among new cases of pulmonary tuberculosis with LC (%)	84.4 (13.5)	79.8 (15.2)	75.1 (16.5)	81.1 (15.1)
Abandonment among new cases of PTB with LC (%)	5.5 (8.9)	10.5 (11.3)	12.1 (13.6)	8.5 (11.1)
**> 300 thousand inhab.**	**n = 166**	**n = 145**	**n = 119**	**n = 430**
	**Mean (SD)**	**Mean (SD)**	**Mean (SD)**	**Mean (SD)**
LC among new cases of pulmonary tuberculosis (%)	75.8 (12.6)	70.8 (14.9)	72.7 (12.7)	73.3 (13.6)
HIV test in new cases of tuberculosis (%)	90.1 (9.1)	87.9 (9.8)	74.7 (17.7)	85.1 (13.9)
DOT among new cases of pulmonary tuberculosis (%)	74.5 (15.8)	21.5 (15.6)	15.4 (14.4)	40.3 (31.3)
Examination of contacts in new cases of tuberculosis (%)	88.5 (11.2)	86.3 (9.3)	56.4 (14.0)	78.9 (18.1)
Cure among new cases of pulmonary tuberculosis with LC (%)	80.7 (8.4)	79.0 (8.4)	74.9 (8.7)	78.5 (8.8)
Abandonment among new cases of pulmonary tuberculosis with LC (%)	8.1 (5.0)	10.0 (6.2)	13.9 (6.4)	10.4 (6.3)

LC: laboratory confirmation; SD: standard deviation; DOT: directly observed treatment; Inhab.: inhabitants; HIV: human immunodeficiency virus.

The municipalities of group A with < 100 thousand inhabitants presented three of the six indicators of tuberculosis with results above the target (laboratory confirmation, contact investigation, and abandonment), and comprised 26.4% of the municipalities and 2% of new cases of the disease. Conversely, the municipalities of groups B and C presented five or all indicators of tuberculosis below the target (at least HIV testing, examination of contacts, DOT, abandonment, and cure), and corresponded to 52.5% of the municipalities and 66.7% of new cases (Tables 1 and 2).

The larger the size, the higher the percentages of laboratory confirmation and HIV testing; however, the greater the abandonment and the lower the DOT (Table 2). For small-sized municipalities (< 50 thousand inhabitants), those classified in group A presented the highest values for laboratory confirmation (mean = 77.4%), DOT (mean = 85.3%), and cure (mean = 87.3 %), whereas the cities in group C presented the lowest percentages for laboratory confirmation (mean = 11.8%) and cure (mean = 0.0%) and DOT below the target (mean = 52.4%) (Figure 2 and Table 2).

In large municipalities (> 300 thousand inhabitants), those classified as group A had abandonment below the target (mean = 8.1%) and HIV testing with a mean of 90.1%, which even below the target (< 100%) was the highest among the 12 identified groups. Conversely, in group C of large municipalities, which includes 19 of the 27 capitals and 43.1% of new cases of tuberculosis, we found the lowest percentages of contact investigation (mean = 56.4%) and DOT (mean = 15.4%). In addition, HIV testing (mean = 74.7%) and abandonment (mean = 13.9%) were below the target (Figure 2 and Table 2).

**Figure 3:**
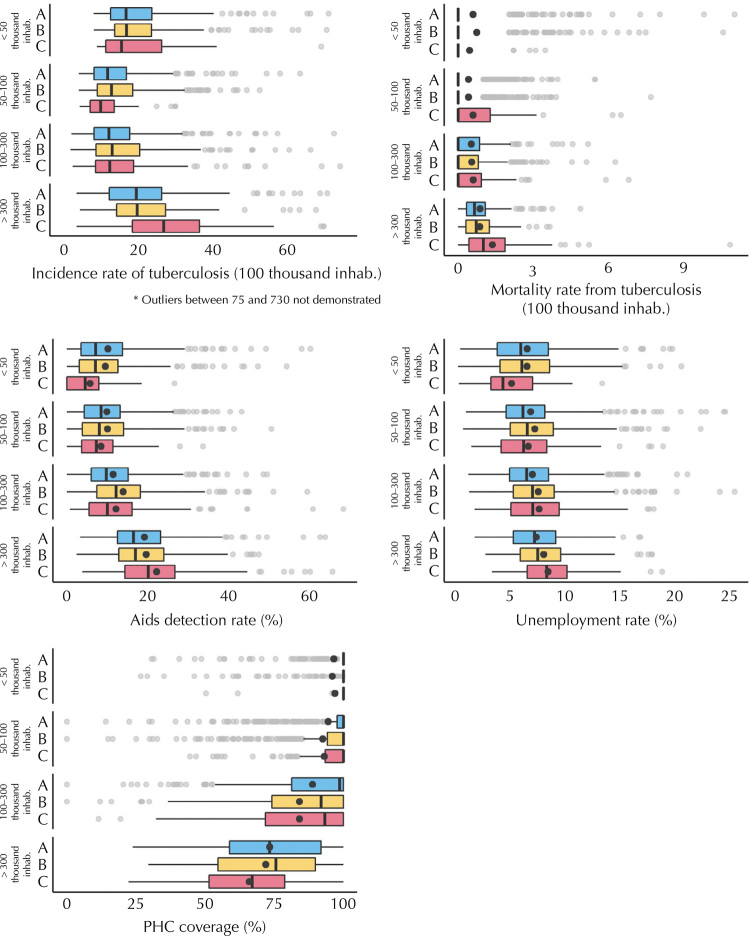
Epidemiological and contextual indicators of tuberculosis, according to population range and groups of performance of operational indicators of the disease, generated by cluster analysis, Brazil, 2015–2018.

Regarding epidemiological and contextual indicators, small municipalities in group C had the lowest rates of Aids detection (median = 4.5/100 thousand inhab.) and tuberculosis mortality (median = 0.0/100 thousand inhab.), lowest unemployment rate (median = 4.4%), and higher coverage of primary health care (median = 100.0%). Conversely, large municipalities in group C showed the highest rates of Aids detection (median = 20.1/100 thousand inhab.) and mortality due to tuberculosis (median = 1.0/100 thousand inhab.), the highest unemployment rate (median = 8.3%), and the lowest coverage of primary health care (median = 67.0%). Furthermore, the incidence of tuberculosis in large municipalities in group C was higher than that found in the small-sized municipalities in group C (median = 27.0/100 thousand inhab. *versus* 15.5/100 thousand inhab.). The general medians for each population range demonstrated that the larger the population, the higher the unemployment rate and the lower the coverage of primary health care (Figure 3).

PHC: primary health care.

In the sensitivity analysis by the LPA method, the use of two to four latent profiles was more adequate, that is, it presented lower values of AIC and BIC, being suggested the one with two profiles. Nevertheless, according to entropy, a measure of the amount of information gathered by the model, and to compare with the results obtained by the k-means method, we adopted three latent profiles for each population range of the municipalities. Among municipalities with < 50 thousand inhabitants, the percentage of agreement of the k-means method in relation to the LPA, that is, the municipalities that were classified in the same groups A, B, or C, was 92.6% (500/540); among those with 50–100 thousand inhabitants, 93.2% (871/935); among those with 100–300 thousand inhabitants, 91.1% (856/940); and among those with > 300 thousand inhabitants, 86.7% (373/430). The final agreement was 91.4% (2,600/2,845).

## DISCUSSION

This study evaluated an important portion of Brazilian municipalities, which were responsible for notifying virtually all new cases of tuberculosis in the studied period (2015–2018). By classifying Brazilian municipalities according to the population range and the operational indicators of the disease, we observed, in most municipalities, an unsatisfactory performance for tuberculosis control. We highlight those with > 300 thousand inhabitants in group C, which notified a high percentage of new cases and presented high rates of incidence and mortality from the disease, detection of Aids, unemployment, and low coverage of primary health care.

Laboratory confirmation was above the target for municipalities that notified a high percentage of new cases of tuberculosis, which demonstrates the progress of this indicator in recent years in the country^2^. Most of the new cases occurred in municipalities where the contact investigation is below the target, which may limit the adequate control of disease transmission from early diagnosis and treatment^10^. Although the national average of HIV testing increased from 65.3% in 2011 to 82.5% in 2019^2^, this indicator was below the target in all groups of municipalities, as also reported in Canada^18^. It is worth highlighting the potential for partnerships between tuberculosis and Aids programs to advance the implementation of rapid HIV testing^19^. The main action to support and monitor tuberculosis treatment^10^, namely DOT, did not reach the target in any group, being the indicator with the highest percentage of non-compliance of our study (19.2%), a difficulty previously pointed out^20^. Abandonment was above the target in few municipalities and cure in none of the groups. It is worth mentioning that these indicators of treatment outcome are sensitive to failures in the implementation of tuberculosis control programs^7^and may be related to the physical distance between patients and the health services that treat the disease^21^.

Large-sized municipalities have higher percentages of laboratory confirmation, HIV testing, and abandonment and lower percentages of contact investigation, DOT, and cure. In these locations, laboratory confirmation is higher, possibly due to the implementation of the *Rede de Teste Rápido para Tuberculose* (Network for Rapid Tuberculosis Testing) in mid-2014^22^ and the wide range of procedures in public healthcare services^23^, which also ensures greater HIV testing. However, larger municipalities had lower coverage of primary health care, which may have repercussions on the lower performance of the contact investigation and DOT and on the higher occurrence of unfavorable treatment outcomes, as primary health care is closer to the residents of people with tuberculosis and can provide them with better treatment support and monitoring^7^.

In this study, municipalities with high coverage of primary health care, such as those in group A with < 100 thousand inhabitants, presented good performance for tuberculosis control. Nevertheless, these municipalities comprised a few of the new cases of the disease. It is worth considering that, within primary health care, units with *Estratégia de Saúde da Família* (ESF – Family Health Strategy) offer regular actions to control tuberculosis and detect more cases of the disease in relation to health services without this strategy^24^.

Conversely, the municipalities with < 50 thousand inhabitants in group C, although with high coverage of primary health care, presented poor performance for tuberculosis control, considering the low rates of laboratory confirmation, HIV testing, and cure, and high abandonment, while having a low incidence of tuberculosis, Aids, and low unemployment rate. In these locations, factors, such as low health budget and poor professional training, probably contribute more significantly to the unsatisfactory performance of tuberculosis control. The stratification of municipalities by population range before their classification by tuberculosis control performance was crucial to obtain more homogeneous results, considering that, as described in a previous study, this performance is not only related to population size^8^. Although larger municipalities generally provide tools to properly operationalize the tuberculosis program^17^, such as greater infrastructure for laboratory confirmation and HIV testing, effective disease control actions can be carried out in smaller locations with limited resources^9^.

It is noteworthy that among the municipalities with > 300 thousand inhabitants in group C, in which a high percentage of new cases of tuberculosis were notified, HIV testing, contact investigation, DOT, abandonment, and cure were below the target. In addition, this group had the lowest coverage of primary health care and the highest rates of unemployment, incidence and mortality from tuberculosis, and detection of Aids. A previous study found an association between the incidence of tuberculosis and unemployment rates and Aids detection^12^. It is worth considering that the high incidence rates of tuberculosis and Aids represent a high risk of infection for these diseases and a higher demand for the health sector^8^. Therefore, in these larger municipalities with unsatisfactory performance for tuberculosis control, disease control initiatives should be prioritized^19^, especially in socioeconomically deprived areas, where mortality rates from the disease are higher^25^ and, due to poor coverage of primary health care, abandonment is high^26^.

Moreover, in larger municipalities, population clusters whose residents have poor health status and greater susceptibility to tuberculosis^27^are common, considering that tuberculosis is a socially determined disease and poverty impacts all its stages, from transmission to treatment outcomes^28^. Cash transfer programs, such as *Bolsa Família* (Family Funding Program), can change this scenario, as they have been related to lower incidence of the disease^29^, higher cure^30^, and lower abandonment^31^.

A previous study evaluated tuberculosis control in Brazilian municipalities^9^. The contribution of our study is the classification of the performance of municipal disease control by its operational indicators, considering the size in virtually all municipalities where cases of the disease were notified in Brazil, thus having high internal validity. In addition, it was innovative in applying a second clustering method, the LPA, in such a way to complement the k-means method and obtain highly similar results, which is probably a unique use and comparison in the field of health.

However, this study has limitations. The first is the use of administrative data, which may have deficiencies in the quality of registration and may underestimate the percentage of contact investigation, DOT, and HIV testing. The second is that the most current values for the unemployment rate of the municipalities derive from the 2010 Demographic Census, which may not portray the reality of the evaluation period of tuberculosis indicators (2015 to 2018). In larger municipalities, the control evaluation should be carried out on an intramunicipal scale, considering its social and economic heterogeneity and the high number of cases of the disease, while in small municipalities this evaluation is also challenging due to the reduced number of cases of the disease, limitations minimized by the segmentation according to population range and analysis of the four-year period. In this study, we considered the process dimensions and results instead of the structure of the tuberculosis control program. Finally, the ecological approach makes it impossible for the results to be extrapolated to the individual level.

## CONCLUSION

In this study, we performed an evaluation of tuberculosis control in Brazilian municipalities based on the operational indicators of the disease, and verified that most new cases are followed up in larger municipalities with difficulties performing adequate control of the disease (that is, with low HIV testing, low contact investigation, low directly observed therapy, low cure, and high abandonment) and with high incidence and mortality from tuberculosis, detection of Aids, high unemployment rate, and low coverage of primary health care. In these locations, the expansion of primary health care and social protection programs can ensure better outcomes for tuberculosis indicators, especially those directly related to availability and access to health professionals such as contact investigation, directly observed therapy, cure, and abandonment.
