# Supplementary material for: Performance evaluation of tuberculosis control in Brazilian municipalities
Source: Rev Saude Publica. 2022 Jun 7;56:53. doi: 10.11606/s1518-8787.2022056004020 (PMC9239336; doi:10.11606/s1518-8787.2022056004020)
Supplement: Translation

# Translation: Avaliação de desempenho do controle da tuberculose em municípios brasileiros

**Authors:** Priscila Fernanda Porto Scaff Pinto, Beatriz Pinheiro Schindler dos Santos, Camila Silveira Silva Teixeira, Joilda Silva Nery, Leila Denise Alves Ferreira Amorim, Mauro Niskier Sanchez, Mauricio Lima Barreto, Julia Moreira Pescarini

**Affiliations:** I Brasil Fundação Oswaldo Cruz. Centro de Integração de Dados e Conhecimentos para a Saúde. Salvador, BA, Brasil; II Brasil Universidade Federal da Bahia. Instituto de Saúde Coletiva. Departamento de Saúde Coletiva I. Salvador, BA, Brasil; III Brasil Universidade Federal da Bahia. Instituto de Matemática. Departamento de Estatística. Salvador, BA, Brasil; IV Brasil Universidade de Brasília. Faculdade de Ciências da Saúde. Departamento de Saúde Coletiva. Brasília, DF, Brasil; V UK London School of Hygiene & Tropical Medicine. Faculty of Epidemiology and Population Health. London, UK

**Keywords:** Tuberculose, prevenção & controle, Avaliação de Processos e Resultados em Cuidados de Saúde, Avaliação de Programas e Projetos de Saúde, Estudos Ecológicos

## Abstract

**OBJETIVO:**

Avaliar o desempenho no controle da tuberculose dos municípios brasileiros.

**MÉTODOS:**

Estudo ecológico com municípios brasileiros que notificaram pelo menos quatro casos novos de tuberculose, com no mínimo um caso novo de tuberculose pulmonar entre 2015 e 2018. Os municípios foram estratificados de acordo com a população em < 50 mil, 50–100 mil, 100–300 mil e > 300 mil habitantes e foi utilizado o método k-médias para agrupá-los dentro de cada faixa populacional segundo desempenho de seis indicadores da doença.

**RESULTADOS:**

Foram incluídos 2.845 municípios brasileiros abrangendo 98,5% (208.007/211.174) dos casos novos de tuberculose do período. Para cada faixa populacional identificou-se três grupos (A, B e C) de municípios segundo desempenho dos indicadores: A os mais satisfatórios, B os intermediários e C os menos satisfatórios. Municípios do grupo A com < 100 mil habitantes apresentaram resultados acima das metas para confirmação laboratorial (≥ 72%), abandono (≤ 5%) e cura (≥ 90%), e abrangeram 2% dos casos novos da doença. Por outro lado, os municípios dos grupos B e C apresentaram pelo menos cinco indicadores com resultados abaixo das metas – testagem HIV (< 100%), exame de contatos (< 90%), tratamento diretamente observado (< 90%), abandono (> 5%) e cura (< 90%) –, e corresponderam a 66,7% dos casos novos de tuberculose. Já no grupo C dos municípios com > 300 mil habitantes, que incluiu 19 das 27 capitais e 43,1% dos casos novos de tuberculose, encontrou-se os menores percentuais de exames de contatos (média = 56,4%) e tratamento diretamente observado (média = 15,4%), elevado abandono (média = 13,9%) e baixa cobertura da atenção básica (média = 66,0%).

**CONCLUSÕES:**

Grande parte dos casos novos de tuberculose ocorreu em municípios com desempenho insatisfatório para o controle da doença, onde expandir a cobertura da atenção básica pode reduzir o abandono e elevar o exame de contatos e tratamento diretamente observado.

## INTRODUÇÃO

O Brasil está entre os trinta países com maior número de casos de tuberculose^1^. Em 2020, foram notificados 66,8 mil casos novos da doença e a incidência foi de 31,6 por 100 mil habitantes, valores inferiores ao esperado devido à pandemia de covid-19, uma vez que em 2019 a incidência foi de 37,4 por 100 mil habitantes e a mortalidade foi de 2,2 por 100 mil habitantes^2^. O Plano Nacional pelo Fim da Tuberculose como Problema de Saúde Pública foi lançado em 2017 com metas para reduzir a incidência para menos de 10/100 mil habitantes e a mortalidade para menos de 1/100 mil habitantes até 2035^3^.

A avaliação e monitoramento dos indicadores de saúde têm reafirmado a importância do Sistema Único de Saúde (SUS) em todos os níveis de atenção^4^. A avaliação de indicadores operacionais da tuberculose orienta a tomada de decisões e direciona ações e políticas de saúde de combate a esse problema de saúde^2^. Em 2019, os indicadores de cura (70,1%) e abandono (12%)^2^ estiveram aquém do almejado para controle da doença^5^, já que a Organização Mundial da Saúde recomenda que a taxa de cura seja de no mínimo 90% e o abandono seja até 5%^6^. Esses desfechos de tratamento podem ser influenciados pela qualidade dos programas de controle da tuberculose^7^.

Avaliação anterior do desempenho das atividades de controle da tuberculose realizada entre 2001 e 2003 identificou que os municípios brasileiros com bom desempenho eram menores em porte populacional, podendo apresentar ou não elevada realização de tratamento diretamente observado (TDO). Já aqueles com desempenho regular e moderado possuíam porte médio ou grande, abandono elevado e maiores incidências para tuberculose e/ou aids^8^. Avaliação recente dos programas de controle da tuberculose incorporou indicadores socioeconômicos, epidemiológicos e operacionais à classificação dos municípios por prioridade de atuação programática do Ministério da Saúde, sugerindo adoção de ações intersetoriais segundo incidência da doença, de acordo com a especificidade de cada realidade local^9^.

Entretanto, as classificações realizadas unicamente por indicadores operacionais da doença permitem avaliar a assistência à tuberculose de forma independente da incidência, o que é útil, pois permite priorizar ações programáticas em escala local, visando controle mais efetivo da doença. Assim, o objetivo deste estudo foi realizar uma avaliação do desempenho dos municípios brasileiros segundo os indicadores operacionais da tuberculose e descrever/investigar sua relação com indicadores epidemiológicos e contextuais entre 2015 e 2018.

## MÉTODOS

### Delineamento do Estudo e Fontes de Informação

Estudo ecológico cujas unidades de análise foram os municípios brasileiros classificados a partir de indicadores operacionais da tuberculose entre 2015 e 2018. Foi escolhido um período de quatro anos, visando suavizar as variações dos indicadores que poderiam ocorrer caso escolhêssemos apenas um ano, a escolha foi feita também levando em consideração que 2018 foi o último ano em que os dados ainda disponibilizavam os desfechos de tratamento. Os registros de tuberculose e aids foram extraídos do Sistema de Informação de Agravos de Notificação (Sinan) (2015–2018) em 17 de outubro de 2020. As estimativas populacionais anuais (2015–2018) e a taxa de desemprego por município entre pessoas ≥ 16 anos para 2010 foram obtidas no Instituto Brasileiro de Geografia e Estatística (IBGE). O percentual de cobertura da atenção básica (AB) provém do Banco e-Gestor da AB (dezembro de 2018).

### População de Estudo

Foram incluídos municípios brasileiros que notificaram ao Sinan pelo menos quatro casos novos de tuberculose no período (2015–2018), sendo no mínimo um caso novo de tuberculose pulmonar, critério utilizado para inserir aqueles que, em média, tenham notificado um caso novo de tuberculose por ano e evitar a inflação de zeros nos dados e cálculo de indicadores com elevada variação devido aos poucos casos. Foi definido como caso novo de tuberculose a pessoa notificada ao Sinan com a doença ativa, independente da classificação clínica, que nunca se submeteu ao tratamento anti-tuberculose ou o fez por menos de 30 dias e, como caso novo de tuberculose pulmonar, a pessoa notificada ao Sinan com a doença ativa, cuja classificação clínica foi pulmonar, ou seja, quando o bacilo acomete os pulmões^10^.

### Variáveis

No período de 2015 a 2018, conjuntamente, foram calculados os onze indicadores operacionais da tuberculose do Plano Nacional pelo Fim da Tuberculose como Problema de Saúde Pública. Para o presente estudo, cinco indicadores foram excluídos por apresentarem baixa abrangência, ou seja, impossibilidade de cálculo para no mínimo 52,8% dos municípios: número de casos de tuberculose notificados como pós-óbito; realização de cultura de escarro entre casos de retratamento de tuberculose pulmonar; realização de teste de sensibilidade entre casos de retratamento de tuberculose pulmonar com cultura positiva; realização de terapia antirretroviral entre casos novos de coinfecção tuberculose -HIV; e cura de casos novos de tuberculose multidrogarresistente. Assim, os seis indicadores avaliados foram: confirmação laboratorial entre casos novos de tuberculose pulmonar; exame entre contatos de casos novos de tuberculose pulmonar com confirmação laboratorial; testagem para o vírus da imunodeficiência humana (HIV) entre casos novos de tuberculose ; TDO entre casos novos de tuberculose pulmonar; abandono do tratamento entre casos novos de tuberculose pulmonar com confirmação laboratorial; e cura de casos novos de tuberculose pulmonar com confirmação laboratorial^5^.

Entre os indicadores epidemiológicos, foram calculadas as médias anuais de incidência e mortalidade por todas as formas de tuberculose e da taxa de detecção de aids. Entre os indicadores contextuais, ou seja, aqueles que podem influenciar a ocorrência da doença e o desempenho de ações programáticas^11^, foram incluídas a taxa de desemprego e a cobertura da atenção básica por se associarem com a incidência de tuberculose em estudos de avaliação anteriores^9,12^.

### Análise

Foi realizada uma análise descritiva dos indicadores operacionais da tuberculose em cada município utilizando o cálculo das médias e respectivos desvios padrão. Optou-se pelas médias por serem a medida de tendência central mais comum para indicadores de tuberculose, por serem próximas das medianas, por considerarem todos os valores do conjunto de dados e por serem comparáveis com o método k-médias e com outras avaliações como o presente estudo. Os municípios foram estratificados nas seguintes faixas populacionais: ≤ 50 mil habitantes; 50 mil a 100 mil habitantes; 100 mil a 300 mil habitantes; e > 300 mil habitantes. Para cada faixa populacional, os municípios foram agrupados de acordo com o desempenho dos indicadores operacionais da tuberculose por meio do método não-hierárquico k-médias, que define a variação total intragrupo como a soma de quadrados das distâncias euclidianas entre os pontos e seus respectivos centroides (within sum of squares – WSS). O método k-médias identifica um número k de grupos com características parecidas, ou seja, cujas distâncias entre as médias de uma variável são mínimas para os membros do mesmo grupo e são máximas em relação aos demais grupos^13^. Foram utilizadas 200 sementes iniciais para garantir a estabilidade dos resultados. O valor de k foi definido pelo método do cotovelo, que constrói um gráfico do WSS de acordo com o número de grupos, permitindo identificar um valor adequado, ou seja, a quantidade de grupos em que os dados podem ser divididos para que os grupos sejam suficientemente distintos entre si^14^.

Os indicadores operacionais da tuberculose foram interpretados conforme cumprimento ou não das metas e/ou recomendações sendo: ≥ 72% para confirmação laboratorial^10^, ≥ 90% para exame de contatos^15^, 100% para testagem HIV^10^, ≥ 90% para realização do TDO^16^, ≤ 5% para abandono^6^ e ≥ 90% para cura^15^.

Após o agrupamento dos municípios, de acordo com o desempenho dos indicadores operacionais da tuberculose, foi realizada uma análise descritiva dos indicadores epidemiológicos da tuberculose e da aids, e dos indicadores contextuais para cada grupo encontrado a partir das médias e respectivos desvios padrão, medianas e respectivos intervalos interquartílicos.

Para testar se os grupos gerados pelo método k-médias eram sensíveis ao método de classificação, os grupos foram reestimados e descritos pela análise de perfis latentes (APL). Adotou-se uma matriz de covariâncias diagonal, com variâncias diferentes por perfis, considerando entre dois e oito perfis latentes. Foram avaliadas as medidas Critério de Informação de Akaike (AIC), Critério de Informação Bayesiano (BIC) e entropia. Os perfis latentes para cada faixa populacional foram caracterizados pelas médias, desvios padrão e matriz de correlações^17^.

As análises foram realizadas no programa R Studio vinculado ao R na versão 4.0.3, com auxílio das bibliotecas tidyverse, factoextra, NbClust e tidyLPA.

### Considerações Éticas

O estudo utilizou dados administrativos de livre acesso e agregados por municípios, portanto isentos de variáveis que possibilitem identificar as pessoas com tuberculose e, por isso, não foi necessária aprovação ética.

## RESULTADOS

Entre 2015 e 2018, 4.643 dos 5.570 municípios brasileiros (83,3%) registraram novos casos de tuberculose. Foram incluídos no estudo 2.845 (61,3%) municípios com pelo menos quatro casos novos de tuberculose e um de tuberculose pulmonar. Dessa forma, o estudo abrangeu 98,5% (208.007 de 211.174) dos casos novos da doença nesse período, dos quais 46,6% ocorreram na Região Sudeste, 25,6% no Nordeste, 11,6% no Sul, 11,5% no Norte e 4,6% no Centro-Oeste.

Os municípios de pequeno porte (< 50 mil habitantes) representaram 19% do total e 2,1% dos casos novos de tuberculose. Aqueles de baixo-médio porte (50 a 100 mil habitantes) (32,9%) incluíram 5,4% dos casos novos, os de médio-grande porte (100 a 300 mil habitantes) (33%) incluíram 12,9% dos casos novos e os de grande porte (> 300 mil habitantes) (15,1%) incluíram 79,6% dos casos novos de tuberculose (Tabela 1).

**Tabela 1:** Distribuição dos municípios e casos novos de tuberculose, segundo faixa populacional e grupos de desempenho dos indicadores operacionais da doença, gerados pela análise de agrupamentos, Brasil, 2015 a 2018.

Faixa populacional	Grupos de desempenho						Total	
	A		B		C			
	Municípios	Casos novos de tuberculose	Municípios	Casos novos de tuberculose	Municípios	Casos novos de tuberculose	Municípios	Casos novos de tuberculose
	n = 1.351	n = 69.236	n = 1.071	n = 43.488	n = 423	n = 95.283	n = 2.845	n = 208.007
	n (%)	n (%)	n (%)	n (%)	n (%)	n (%)	n (%)	n (%)
< 50 mil hab.	292 (21,6)	2.667 (3,9)	218 (20,4)	1.570 (3,6)	30 (7,1)	138 (0,14)	540 (19,0)	4.375 (2,1)
50–100 mil hab.	460 (34,0)	5.888 (8,5)	375 (35,0)	4.459 (10,3)	100 (23,6)	927 (1,0)	935 (32,9)	11.274 (5,4)
100–300 mil hab.	433 (32,1)	11.819 (17,1)	333 (31,1)	10.417 (24,0)	174 (41,1)	4.616 (4,8)	940 (33,0)	26.852 (12,9)
> 300 mil hab.	166 (12,3)	48.862 (70,6)	145 (13,5)	27.042 (62,2)	119 (28,1)	89.602 (94,0)	430 (15,1)	165.506 (79,6)

Para cada faixa populacional, o método do cotovelo indicou uma classificação com número de grupos variando entre dois e quatro. Assim, optou-se por três grupos que foram denominados como A, B e C (Figura 1). De forma geral, os municípios do grupo A obtiveram indicadores mais satisfatórios, os do grupo B intermediários e os do grupo C menos satisfatórios, fixando-se o porte do município. A confirmação laboratorial foi acima da meta (≥ 72%) em todos os municípios dos grupos A e nos municípios com > 300 mil habitantes do grupo C (45,2% dos casos novos). O exame de contatos foi acima da meta (≥ 90%) nos grupos A dos municípios com até 300 mil habitantes (33,3% dos casos novos). A testagem HIV, o TDO e a cura não alcançaram a meta (< 100%, < 90% e < 90%, respectivamente) em nenhum grupo. O abandono foi acima da meta (≤ 5%) apenas no grupo A dos municípios com < 100 mil habitantes (4,1% dos casos novos) (Figura 2 e Tabela 2).

**Figura 1:** Distribuição dos municípios brasileiros incluídos no estudo, segundo faixa populacional e os grupos de desempenho dos indicadores operacionais da tuberculose, gerados pela análise de agrupamentos, Brasil, 2015 a 2018.

Nota: a área cinza corresponde aos municípios incluídos no estudo.

**Figura 2:** Descrição dos indicadores operacionais da tuberculose, segundo faixa populacional e grupos de desempenho dos indicadores operacionais da doença, gerados: a) pela análise de agrupamentos (k-médias); e b) pelo método análise de perfis latentes (APL), Brasil, 2015 a 2018.

Hab.: habitantes; HIV: vírus da imunodeficiência humana; TDO: tratamento diretamente observado.

**Tabela 2:** Descrição dos indicadores operacionais da tuberculose, segundo faixa populacional e grupos de desempenho dos indicadores operacionais, gerados pela análise de agrupamentos, Brasil, 2015 a 2018.

Indicadores operacionais da tuberculose segundo faixa populacional	Grupos de desempenho			Total n = 540
	A	B	C	
< 50 mil hab.	n = 292	n = 218	n = 30	
	Média (DP)	Média (DP)	Média (DP)	Média (DP)
CL entre casos novos de tuberculose pulmonar (%)	77,4 (22,9)	69,5 (25,1)	11,8 (21,9)	70,6 (28,0)
Teste HIV em casos novos de tuberculose (%)	80,3 (23,1)	70,3 (27,3)	68,5 (33,5)	75,6 (26,0)
TDO em casos novos de tuberculose pulmonar (%)	85,3 (16,3)	21,4 (18,3)	52,5 (38,3)	57,7 (36,1)
Exame de contatos em casos novos de tuberculose (%)	91,9 (13,6)	75,3 (26,7)	80,7 (30,4)	84,6 (22,4)
Cura entre casos novos de tuberculose pulmonar com CL (%)	87,3 (18,0)	82,2 (21,5)	0,0 (0,0)	80,4 (27,4)
Abandono entre casos novos de tuberculose pulmonar com CL (%)	4,5 (11,3)	6,5 (13,4)	9,2 (26,7)	5,6 (13,5)
**50 a 100 mil hab.**	**n = 460**	**n = 375**	**n = 100**	**n = 935**
	**Média (DP)**	**Média (DP)**	**Média (DP)**	**Média (DP)**
CL entre casos novos de tuberculose pulmonar (%)	77,0 (19,1)	69,6 (21,0)	46,7 (24,9)	70,8 (22,5)
Teste HIV em casos novos de tuberculose (%)	83,0 (19,0)	71,6 (24,9)	71,2 (25,6)	77,2 (23,0)
TDO em casos novos de tuberculose pulmonar (%)	81,8 (16,3)	20,4 (17,6)	43,3 (28,8)	53,1 (34,5)
Exame de contatos entre casos novos de tuberculose (%)	92,0 (13,8)	81,3 (20,6)	80,3 (22,2)	86,4 (18,6)
Cura entre casos novos de tuberculose pulmonar com CL (%)	87,1 (14,6)	86,4 (14,5)	39,1 (24,6)	81,7 (21,7)
Abandono entre casos novos de tuberculose pulmonar com CL (%)	4,2 (8,3)	5,3 (9,2)	25,8 (28,8)	7,0 (14,1)
**100 a 300 mil hab.**	**n = 433**	**n = 333**	**n = 174**	**n = 940**
	**Média (DP)**	**Média (DP)**	**Média (DP)**	**Média (DP)**
CL entre casos novos de tuberculose pulmonar (%)	73,5 (17,5)	71,6 (17,8)	64,9 (19,8)	71,2 (18,3)
Teste HIV em casos novos de tuberculose (%)	85,5 (17,1)	86,6 (13,1)	58,0 (20,2)	80,8 (19,7)
TDO em casos novos de tuberculose pulmonar (%)	79,6 (16,2)	16,9 (15,6)	23,0 (19,6)	46,9 (34,6)
Exame de contatos entre casos novos de tuberculose (%)	91,9 (11,3)	89,3 (10,9)	58,2 (22,1)	84,7 (18,8)
Cura entre casos novos de tuberculose pulmonar com CL (%)	84,4 (13,5)	79,8 (15,2)	75,1 (16,5)	81,1 (15,1)
Abandono entre casos novos de tuberculose pulmonar com CL (%)	5,5 (8,9)	10,5 (11,3)	12,1 (13,6)	8,5 (11,1)
**> 300 mil hab.**	**n = 166**	**n = 145**	**n = 119**	**n = 430**
	**Média (DP)**	**Média (DP)**	**Média (DP)**	**Média (DP)**
CL entre casos novos de tuberculose pulmonar (%)	75,8 (12,6)	70,8 (14,9)	72,7 (12,7)	73,3 (13,6)
Teste HIV em casos novos de tuberculose (%)	90,1 (9,1)	87,9 (9,8)	74,7 (17,7)	85,1 (13,9)
TDO em casos novos de tuberculose pulmonar (%)	74,5 (15,8)	21,5 (15,6)	15,4 (14,4)	40,3 (31,3)
Exame de contatos entre casos novos de tuberculose (%)	88,5 (11,2)	86,3 (9,3)	56,4 (14,0)	78,9 (18,1)
Cura entre casos novos de tuberculose pulmonar com CL (%)	80,7 (8,4)	79,0 (8,4)	74,9 (8,7)	78,5 (8,8)
Abandono entre casos novos de tuberculose pulmonar com CL (%)	8,1 (5,0)	10,0 (6,2)	13,9 (6,4)	10,4 (6,3)

CL: confirmação laboratorial; DP: desvio padrão; TDO: tratamento diretamente observado; Hab.: habitantes; HIV: vírus da imunodeficiência humana.

Os municípios do grupo A com < 100 mil habitantes apresentaram três dos seis indicadores de tuberculose com resultados acima da meta (confirmação laboratorial, exame de contatos e abandono), e abrangeram 26,4% dos municípios e 2% dos casos novos da doença. Por outro lado, os municípios dos grupos B e C apresentaram cinco ou todos os indicadores de tuberculose abaixo da meta (ao menos testagem HIV, exame de contatos, TDO, abandono e cura), e corresponderam a 52,5% dos municípios e 66,7% dos casos novos (Tabelas 1 e 2).

Quanto maior o porte, maiores os percentuais de confirmação laboratorial e testagem HIV, entretanto, maior o abandono e menor o TDO (Tabela 2). Para os municípios pequenos (< 50 mil habitantes), aqueles classificados no grupo A apresentaram os valores mais elevados para confirmação laboratorial (média = 77,4%), TDO (média = 85,3%) e cura (média = 87,3%), enquanto as cidades do grupo C apresentaram os menores percentuais para confirmação laboratorial (média = 11,8%) e cura (média = 0,0%) e TDO abaixo da meta (média = 52,4%) (Figura 2 e Tabela 2).

Já nos municípios de grande porte (> 300 mil habitantes), aqueles classificados como grupo A tiveram abandono abaixo da meta (média = 8,1%) e testagem HIV com média de 90,1%, que mesmo abaixo da meta (< 100%) foi o maior entre os 12 grupos identificados. Já no grupo C dos municípios de grande porte, que inclui 19 das 27 capitais e 43,1% dos casos novos de tuberculose, foram encontrados os menores percentuais de exame de contatos (média = 56,4%) e TDO (média = 15,4%). Além disso, testagem HIV (média = 74,7%) e abandono (média = 13,9%) foram abaixo da meta (Figura 2 e Tabela 2).

Com relação aos indicadores epidemiológicos e contextuais, os municípios de pequeno porte do grupo C apresentaram as menores taxas de detecção de aids (mediana = 4,5/100 mil hab.) e de mortalidade por tuberculose (mediana = 0,0/100 mil hab.), a menor taxa de desemprego (mediana = 4,4%) e a maior cobertura da atenção básica (mediana = 100,0%). Já os municípios de grande porte do grupo C apresentaram as maiores taxas de detecção de aids (mediana = 20,1/100 mil hab.) e de mortalidade por tuberculose (mediana = 1,0/100 mil hab.), a maior taxa de desemprego (mediana = 8,3%) e a menor cobertura da atenção básica (mediana = 67,0%). Além disso, a incidência de tuberculose nos municípios de grande porte do grupo C foi superior à encontrada nos municípios do grupo C de menor porte (mediana = 27,0/100 mil hab. *versus* 15,5/100 mil hab. As medianas gerais para cada faixa populacional revelaram que, quanto maior a população, maior a taxa de desemprego e menor a cobertura da atenção básica (Figura 3).

**Figura 3:** Indicadores epidemiológicos da tuberculose e de contexto, segundo faixa populacional e os grupos de desempenho dos indicadores operacionais da doença, gerados pela análise de agrupamentos, Brasil, 2015 a 2018.

AB: atenção básica.

Na análise de sensibilidade pelo método APL, a utilização de dois a quatro perfis latentes se mostrou mais adequada, ou seja, apresentou menores valores de AIC e BIC, sendo sugerido aquele com dois perfis. No entanto, por meio da entropia, uma medida da quantidade de informação capturada pelo modelo, e para comparar com os resultados obtidos pelo método k-médias, adotou-se três perfis latentes para cada faixa populacional dos municípios. Entre municípios < 50 mil habitantes o percentual de concordância do método k-médias em relação ao APL, ou seja, que foram classificados nos mesmos grupos A, B ou C, foi de 92,6% (500/540); entre os de 50–100 mil habitantes de 93,2% (871/935); entre os de 100–300 mil habitantes de 91,1% (856/940); e entre os com > 300 mil habitantes de 86,7% (373/430). A concordância final foi de 91,4% (2.600/2.845).

## DISCUSSÃO

Esse estudo avaliou uma parcela importante dos municípios brasileiros, que foram responsáveis por notificar praticamente todos os casos novos de tuberculose no período estudado (2015–2018). Ao classificar os municípios brasileiros de acordo com a faixa populacional e segundo os indicadores operacionais da doença, observamos, em grande parte dos municípios, um desempenho insatisfatório para o controle da tuberculose. Destacando-se aqueles com > 300 mil habitantes do grupo C, que notificaram elevado percentual dos casos novos e apresentaram altas taxas de incidência e mortalidade pela doença, detecção de aids, desemprego e baixa cobertura da atenção básica.

A confirmação laboratorial foi acima da meta para os municípios que notificaram alto percentual dos casos novos de tuberculose, o que demonstra o avanço desse indicador nos últimos anos no país^2^. Grande parte dos casos novos ocorreram em municípios em que o exame de contatos se encontra abaixo da meta, o que pode limitar o controle adequado da transmissão da doença a partir do diagnóstico e tratamento precoce^10^. Embora a média nacional de testagem HIV tenha melhorado de 65,3% em 2011 para 82,5% em 2019^2^, esse indicador foi abaixo da meta em todos os grupos de municípios assim como encontrado no Canadá^18^. É importante destacar o potencial de parcerias entre os programas de tuberculose e aids para avançar na implantação do teste rápido de HIV^19^. O TDO, principal ação de apoio e monitoramento do tratamento da tuberculose^10^, não alcançou a meta em nenhum grupo, sendo o indicador com maior percentual de não preenchimento do presente estudo (19,2%), dificuldade já apontada anteriormente^20^. O abandono foi acima da meta em poucos municípios e a cura em nenhum grupo. Vale destacar que esses indicadores de desfecho de tratamento são sensíveis às falhas na execução dos programas de controle da tuberculose^7^e podem estar relacionadas à distância física existente entre pacientes e os serviços de saúde que tratam a doença^21^.

Municípios de grande porte possuem maiores percentuais de confirmação laboratorial, testagem HIV e abandono e menores percentuais de exame de contatos, TDO e cura. Nesses locais, a confirmação laboratorial é maior, possivelmente devido à implantação da Rede de Teste Rápido para Tuberculose em meados de 2014^22^ e à ampla oferta de procedimentos nos serviços públicos^23^, que também garante maior testagem HIV. Entretanto, municípios maiores apresentaram menor cobertura da atenção básica, o que pode repercutir na menor realização de exame de contatos e de TDO e na maior ocorrência de desfechos desfavoráveis de tratamento uma vez que a atenção básica está mais próxima dos domicílios das pessoas com tuberculose e pode fornecer a elas apoio e monitoramento do tratamento melhores^7^.

Neste estudo, municípios com elevadas coberturas da atenção básica, como os do grupo A com < 100 mil habitantes apresentaram bom desempenho para o controle da tuberculose. Entretanto, esses municípios abrangeram pequena parte dos casos novos da doença. Vale considerar que, dentro da atenção básica, as unidades com Estratégia de Saúde da Família (ESF) oferecem ações regulares de controle da tuberculose e detectam mais casos da doença em relação aos serviços de saúde sem essa estratégia^24^.

Por outro lado, os municípios com < 50 mil habitantes do grupo C, ainda que tenham elevada cobertura da atenção básica, apresentaram desempenho insatisfatório para o controle da tuberculose, haja vista as baixas taxas de confirmação laboratorial, testagem HIV e cura, e elevado abandono, ao mesmo tempo em que possuem baixa incidência de tuberculose, aids e baixa taxa de desemprego. Nesses locais, é possível que existam fatores como baixo orçamento para saúde e pouco treinamento profissional, contribuindo de forma mais significativa para o desempenho insatisfatório do controle da tuberculose. A estratificação dos municípios por faixa populacional antes de sua classificação por desempenho do controle da tuberculose foi crucial para obtenção de resultados mais homogêneos, já que, como descrito em estudo anterior, tal desempenho não está unicamente relacionado ao tamanho populacional^8^. Embora os municípios maiores geralmente disponham de instrumentos para operacionalizar adequadamente o programa de tuberculose^17^, como maior infraestrutura para confirmação laboratorial e testagem HIV, é possível executar ações efetivas de controle da doença em localidades menores com recursos limitados^9^.

Destaca-se que, entre os municípios com > 300 mil habitantes do grupo C, em que foi notificado elevado percentual dos casos novos de tuberculose, a testagem HIV, o exame de contatos, o TDO, o abandono e a cura foram abaixo da meta. Além disso, esse grupo apresentou as menores coberturas da atenção básica e as mais elevadas taxas de desemprego, incidência e mortalidade por tuberculose e detecção de aids. Estudo anterior encontrou associação entre a incidência de tuberculose e as taxas de desemprego e de detecção de aids^12^. Vale considerar que as elevadas taxas de incidência de tuberculose e de aids representam alto risco de infecção para essas doenças e maior demanda para o setor saúde^8^. Sendo assim, nesses municípios maiores com desempenho insatisfatório para o controle da tuberculose, as ações de controle da doença devem ser priorizadas^19^, especialmente nas áreas socioeconomicamente desfavoráveis, em que as taxas de mortalidade pela doença são maiores^25^ e, devido à escassa cobertura da atenção básica, o abandono é alto^26^.

Ademais, em municípios maiores, são comuns os aglomerados populacionais cujos residentes possuem situação de saúde precária e maior suscetibilidade à tuberculose^27^, uma doença socialmente determinada já que a pobreza impacta todos os seus estágios, desde a transmissão até os desfechos de tratamento^28^. Programas de transferência de renda, como o Bolsa Família, podem mudar esse cenário, uma vez que foram relacionados com menor incidência da doença^29^, maior cura^30^ e menor abandono^31^.

Estudo anterior avaliou o controle da tuberculose nos municípios brasileiros^9^. A contribuição do presente estudo é a classificação do desempenho do controle municipal da doença por seus indicadores operacionais, considerando o porte em praticamente todos os municípios onde houve notificação de casos da doença no Brasil, possuindo, assim, alta validade interna. Além disso, inovou em aplicar um segundo método de agrupamento, o APL, de forma complementar ao método k-médias, e obter resultados altamente similares, utilização e comparação provavelmente únicas no campo da saúde.

Contudo, este estudo apresenta limitações. A primeira é a utilização de dados administrativos, que podem ter deficiências na qualidade de registro, podendo subestimar o percentual de exame de contatos, TDO e testagem HIV. A segunda é que os valores mais atuais para taxa de desemprego dos municípios provêm do Censo Demográfico de 2010, o que pode não retratar a realidade do período de avaliação dos indicadores de tuberculose (2015 a 2018). Em municípios maiores, a avaliação do controle deveria ser realizada em escala intramunicipal, tendo em vista sua heterogeneidade social e econômica e elevado número de casos da doença, já em municípios pequenos essa avaliação também é desafiadora, devido ao reduzido número de casos da doença, limitações minimizadas pela segmentação por faixa populacional e análise do período de quatro anos. Neste estudo foram consideradas as dimensões de processo e resultados e não a estrutura do programa de controle da tuberculose. Por último, a abordagem ecológica impossibilita que os resultados sejam extrapolados para o nível individual.

## CONCLUSÕES

No presente estudo, realizamos uma avaliação do controle da tuberculose nos municípios brasileiros a partir dos indicadores operacionais da doença e encontramos que grande parte dos casos novos são acompanhados em municípios maiores e com dificuldades para desempenhar o controle adequado da doença (ou seja, com baixa testagem HIV, baixo exame de contatos, baixo tratamento diretamente observado, baixa cura e elevado abandono) e com elevadas incidência e mortalidade por tuberculose, detecção de aids, alta taxa de desemprego e baixa cobertura da atenção básica. Nesses locais, a expansão da atenção básica e dos programas de proteção social podem garantir melhores resultados para os indicadores da tuberculose, principalmente aqueles diretamente relacionados à disponibilidade e acesso a profissionais de saúde, como o exame de contatos, tratamento diretamente observado, cura e abandono.
